# Requalification of a Brazilian *Trichoderma* Collection and Screening of Its Capability to Decolourise Real Textile Effluent

**DOI:** 10.3390/ijerph14040373

**Published:** 2017-04-01

**Authors:** Dianny Silva Lisboa, Cledir Santos, Renan N. Barbosa, Oliane Magalhães, Laura M. Paiva, Keila A. Moreira, Nelson Lima, Cristina M. Souza-Motta

**Affiliations:** 1 Department of Mycology, Centre of Biological Sciences, Federal University of Pernambuco, Recife, PE 50740-600, Brazil; dy_carol@hotmail.com (D.S.L.); renan.rnb@gmail.com (R.N.B.); olimicomed@yahoo.com.br (O.M.); mesquitapaiva@terra.com.br (L.M.P); cristina.motta@ufpe.br (C.M.S.-M.); 2 Department of Chemical Sciences and Natural Resources, Centro de Excelencia en Investigación Biotecnológica Aplicada al Medio Ambiente (CIBAMA), Scientific and Technological Bioresource Nucleus (BIOREN), Universidad de La Frontera, Temuco 4811-230, Chile; 3 Academic Unity of Garanhuns, Federal Rural University of Pernambuco, Garanhuns, PE 55292-270, Brazil; moreiralab@yahoo.com.br; 4 CEB-Centre of Biological Engineering, Micoteca da Universidade do Minho, University of Minho, Braga 4710-057, Portugal; nelson@ie.uminho.pt

**Keywords:** culture collection, filamentous fungi, laccase, lignin peroxidase, manganese peroxidase, textile effluent decolourisation, *Trichoderma* identification

## Abstract

Water contamination with large amounts of industrial textile coloured effluents is an environmental concern. For the treatment of textile effluents, white-rot fungi have received extensive attention due to their powerful capability to produce oxidative (e.g., ligninolytic) enzymes. In addition, other groups of fungi, such as species of *Aspergillus* and *Trichoderma*, have also been used for textile effluents treatment. The main aim of the present study was to requalify a Brazilian *Trichoderma* culture collection of 51 *Trichoderma* strains, isolated from different sources in Brazil and preserved in the oldest Latin-American Fungal Service Culture Collection, The Micoteca URM WDCM 804 (Recife, Brazil). Fungal isolates were re-identified through a polyphasic approach including macro- and micro-morphology and molecular biology, and screened for their capability to decolourise real effluents collected directly from storage tanks of a textile manufacture. *Trichoderma atroviride* URM 4950 presented the best performance on the dye decolourisation in real textile effluent and can be considered in a scale-up process at industrial level. Overall, the potential of *Trichoderma* strains in decolourising real textile dye present in textile effluent and the production of the oxidative enzymes Lac, LiP and MnP was demonstrated. Fungal strains are available in the collection e-catalogue to be further explored from the biotechnological point of view.

## 1. Introduction

Textile industries waste large water quantities that in some cases return to environment as untreated or incorrectly treated effluents [1]. Due to the presence of non-fixed dyes during the dyeing process, these wastewaters are highly coloured [2,3]. Water contamination with large amounts of coloured effluents is a serious environmental problem.

There are mainly three types of treatment for textile effluents: physical, chemical and biological. In recent years, relevant methods based on biological textile effluents degradation have been developed using bacteria and fungi in aerobic and anaerobic processes [1,2,4,5,6,7,8,9,10]. A recent review on current technologies for biological treatment of textile wastewater discusses in deep these bioprocesses [11]. The use of oxidative enzymes, mainly Lac and peroxidases, in textile industry has increased quickly. It has been due to both: (a) the ability of these oxidative enzymes to bleach textiles; and (b) the biological treatments involving these enzymes seem to be an attractive solution mainly because most existing treatments (e.g., coagulation/flocculation, adsorption, ion exchange, and electrochemical methods) of dye wastewater utilize ineffective and uneconomical processes [12]. In addition, other biomimetic oxidative bleaches, like metalloporphyrins, are very promising catalysts for synthetic textile dyes. However, they also show some limitations and drawbacks [13].

For the treatment of textile effluents, white-rot fungi have been extensive assessed due to their powerful capability to produce oxidative, including ligninolytic (e.g., laccase (Lac), lignin peroxidase (LiP) and manganese peroxidase (MnP)) enzymes [6]. Other groups of fungi have also been evaluated for textile effluents treatment (e.g., species of ascomycetes like *Aspergillus* and *Trichoderma*) [6,11]. In addition, although the oxidative system is frequently produced during fungal secondary metabolism, different microorganisms produce different enzymes depending on cultivation conditions [4,5,6,14].

*Trichoderma* is a very genetically diverse genus with interesting features, which make some of its species of agricultural and industrial interest. Species of *Trichoderma* are widely used in the production of enzymes for both pulp treatment and food production [15]. In addition, there are also different reports showing the potential of these fungi to remediate the pollution of soil and water [14,16,17,18].

All these features make *Trichoderma* an important fungal taxonomic group for biotechnological application. As a matter of consequence, for microbial culture collections that have *Trichoderma* in their catalogues it is important to have these assets identified and characterised according to the current state-of-the-art. Considering this, the main aim of the present study was to requalify a Brazilian *Trichoderma* culture collection of 51 *Trichoderma* strains, isolated from different sources in Brazil and preserved in the oldest Latin-American Fungal Service Culture Collection, The Micoteca URM WDCM 804 (Recife, Brazil). The requalification work was based on the fungal identification through a polyphasic approach including macro- and micro-morphology, molecular biology and screening their capability to decolourise real effluents collected directly from storage tanks of a textile manufacture.

## 2. Materials and Methods

### 2.1. Microorganisms and Culture Conditions

Fifty-one *Trichoderma* strains previously isolated from different substrates of geographic regions of Brazil (see Table 1) were obtained from the Fungal Culture Collection Micoteca URM (Department of Mycology, Federal University of Pernambuco-UFPE, Recife, Brazil, www.ufpe.br/micoteca). The Micoteca URM is a culture collection registered at the World Federation for Culture Collections (WFCC) under the number WDCM 604 and certified in the ISO 9001:2015 standard under the Certificate Number BR018207-1. Fungal strains were obtained from preserved form under mineral oil, revived according to the instructions issued by the Micoteca URM culture collection, and grown and maintained in Potato Dextrose Agar plates (PDA, for 1 L water: 4 g potato starch, 20 g dextrose and 15 g agar).

### 2.2. Morphological Fungal Identification

In order to confirm the taxonomical identification of the 51 *Trichoderma* strains, macro- and micro-morphological analyses were performed (see Table 1). For macro-morphological analyses, the strains were grown on PDA plates at 30, 35 and 40 °C, and on Synthetic Low-Nutrient Agar plates (SNA, for 1 L water: 1.0 g KH_2_PO_4_, 1.0 g KNO_3_, 0.5 g MgSO_4_·7H_2_O, 0.5 g KCl, 0.2 g glucose, 0.2 g sucrose and 20 g agar) at 35 °C, in the dark without humidity control and under aerobic conditions.

For micro-morphological analysis, strains were inoculated on Cornmeal Agar Medium supplemented with dextrose plates (CMD, for 1 L water: 30 g cornmeal, 20 g dextrose and 20 g agar). Fungi were incubated at 25 °C for 3 days in the same conditions described above. Afterwards, 6 mm diameter plugs of each CMD fungal culture were inoculated on PDA plates and grown for 4 days in the same conditions but with a regime of 12:12 h light:dark.

Conidia and phialides, presence of chlamydospores and sterile hyphae were observed using 90% lactic acid slides on a light microscopy. Final morphological identification followed the taxonomic keys and guides available for *Trichoderma* [19,20].

### 2.3. Molecular Fungal Identification

For the molecular identification, the fungal strains were cultivated in Minimum Liquid Medium (MLM, for 1 L water: 10 g glucose, 10 g KH_2_PO_4_; 6 g (NH_4_)_2_SO_4_, 1 g MgSO_4_·7H_2_O, 5 mg FeSO_4_·7H_2_O, 1.6 mg MnSO_4_·H_2_O, 1.4 mg ZnSO_4_·7H_2_O and, 2 mg CaC1_2_·2H_2_O) at 28 °C under static condition for 96 h. Mycelia were filtered and collected for extraction of genomic DNA according to the method previously described elsewhere [21]. Amplifications of gene regions ITS1-5.8S-ITS2 and translation elongation factor 1-alpha (*tef1*) were performed in 50 µL mixture containing: buffer Taq DNA polymerase 1×; 1.5 mM of MgCl_2_; 0.4 μM each primer (ITS1: 5’-TCCGTAGGTGAACCTGCGG-3’; ITS4: 5’-TCCTCCGCTTATTGATATGC-3’; EF1-728F: 5’-CATCGAGAAGTTCGAGAAGG-3’; TEF1 rev: 5’-GCCATCCTTGGAGATACCAGC-3’) [22,23,24]; 0.2 mM of dNTPs; 0.2 U of Taq DNA polymerase; and 25 ng of DNA template.

The thermal cycling parameters consisted of initial denaturation at 94 °C for 2 min followed by 30 cycles of denaturation at 94 °C for 30 s, primer annealing at 55 °C for 30 s, primer extension at 72 °C for 1 min and a final extension for 10 min at 72 °C [20,21]. Amplification products were visualised on 1% agarose gel electrophoresis, stained with 0.5 μg/mL of GelGreen^TM^ under UV light and then photographed and purified using Fermentas^®^ kit (Cambridge, UK). The amplified DNA fragments were sequenced in the Sequencing Platform of the Central Laboratory of the Centre of Biological Sciences, Federal University of Pernambuco, Recife, Brazil.

Electropherograms were analysed and edited in the software Staden Package 2.0. Sequences obtained were used to search the most similar sequences deposited in the GenBank by using the Basic Local Alignment Search Tool (BLAST). The experimental sequences of ITS1-5.8S-ITS2 and *tef1* gene regions were aligned and edited together with the retrieved from the database using the software MEGA 5.0. The phylogenetic tree was constructed using the Neighbour Joining method and the Maximum Parsimony (MP), with 1000 bootstrap resampling using the software MEGA 5.0 [25].

### 2.4. Decolourisation Assay

Samples of textile effluent were collected directly from storage tanks in a textile manufacture at the municipality of Toritama, State of Pernambuco, Brazil. The effluent samples were kept at 4 °C and transferred to the Laboratory of Environmental and Quality Engineering (Department of Chemical Engineering, Federal University of Pernambuco, Recife, Brazil) to perform the physical-chemical analyses and decolourisation assay.

The physical-chemical analyses involved the Chemical Oxygen Demand (COD) by colorimetric method, Biochemical Oxygen Demand (BOD) by azide modification of a Winkler titration method, Colour (HAZEN scale), Turbidity (NTU scale), pH using the potentiometric measurement principle, and Sedimentable Solids (SD) by gravimetric through a 0.45 μm filter membrane, using the standard methods for the examination of water and wastewater [26].

The screening of textile dye decolourisation by *Trichoderma* strains was assessed by using 6 mm diameter plugs of fungal cultures previously grown at 30 °C for 7 days on PDA plates. Plugs were transferred to 125 mL Erlenmeyer flasks containing 30 mL of Malt Broth (MB, for 1 L water: 20 g malt extract, 1 g peptone and 20 g dextrose) supplemented with 1.9 × 10^−2^ mM Indigo Carmine dye (Sigma-Aldrich, St. Louis, MO, USA).

Fungal cultures were incubated in the dark at 28 °C for 8 days. Fungal biomass was then removed by filtration through a 0.45 µm Millipore membrane and absorbance of filtrates was assessed in a Hitachi U-5100 spectrophotometer at 650 nm which is the maximum absorption of Indigo Carmine (ε_650 nm_ = 3.1 × 10^3^ M^−1^·cm^−1^). The Indigo Carmine was used as standard and MB free of inoculum containing dye was used as abiotic control. The decolourisation percentage of Indigo Carmine dye was calculated according to Miranda et al. [27].

Previous to evaluate the decolourisation of the dye present on the real textile effluent, this was sterilised by autoclave at 121 °C during 15 min. Three different experiments were performed. In each case, 3 plugs of 6 mm diameter of fungal cultures grown for 7 days at 30 °C on PDA plates were added to 125 mL Erlenmeyer flasks containing 30 mL of: (1) textile effluent; (2) textile effluent supplemented with 0.5% (*w*/*v*) wheat bran, and (3) textile effluent supplemented with 0.05% (*w*/*v*) extract yeast. Cultures were incubated under static conditions, in the dark at 28 °C for 8 days. Afterwards, 2 mL aliquots were transferred into tubes and centrifuged at 1035 g for 15 min at 4 °C. Supernatants were analysed at 650 nm in a Hitachi U-5100 spectrophotometer. 

The decolourisation results were submitted to analysis of variance and means compared by Friedman test at 5% probability in the ASSISTAT software [28].

### 2.5. Determination of Enzymatic Activities

In order to determine the activity of Lac, LiP and MnP, 6 mm diameter plugs of fungal cultures previously grown for 7 days at 30 °C on PDA plates were transferred into 125 mL Erlenmeyer flasks containing 50 mL of Basal Culture Medium (BCM, for 1 L water: 0.1 g glucose; 0.15 g yeast extract; 4.5 g wheat bran; and 0.05 g NH_4_Cl, pH 6.0). Fungal incubation occurred under static condition in the dark, at 28 °C for 8 days. The biomass was then separated by filtration through a 0.45 µm Millipore membrane. In order to determine the activity of each enzyme, absorbance of filtrates was assessed in a spectrophotometer Hitachi U-5100 and one unit (U) of enzyme was defined as the release of 1.0 µmol product formed per min under the assay conditions.

Lac activity was measured from the oxidation of 2,2′-azino-bis(3-ethylbenzothiazoline-6-sulphonic acid) (ABTS). For this reaction, a mixture containing 0.8 mL ABTS (0.03% *v*/*v*), 0.1 mL sodium acetate buffer (0.1M, pH 5.0) and 0.1 mL enzymatic extract was used [6]. The oxidation of ABTS was determined at 420 nm with ε_420 nm_ = 3.6 × 10^4^ M^−1^·cm^−1^. LiP activity was assessed through the oxidation of veratryl alcohol. For this reaction, a mixture containing 1 mL sodium tartrate buffer solution (125 mM, pH 3.0), 500 µL veratryl alcohol (10 mM), 500 µL hydrogen peroxide (2 mM) and 500 µL enzymatic extract was used. The reaction was started by adding hydrogen peroxide to the mixture and the production of veratraldehyde was determined at 310 nm [29] with ε_310 nm_ = 9.3 × 10^3^ M^−1^·cm^−1^.

MnP activity was assessed by using the methodology of phenol red oxidation measurement as previously described elsewhere [30]. Briefly, the reaction mixture was composed of 500 µL enzymatic extract, 100 µL phenol red, 100 µL sodium lactate (250 mM), 200 µL bovine albumin (0.5% *w*/*v*), 50 µL sulphate manganese (2 mM), 50 µL hydrogen peroxide (2 mM) and 1.0 mL sodium succinate buffer (20 mM, pH 4.0). The reactions were performed at 30 °C for 5 minutes and were stopped by adding 2 N NaOH (40 µL). MnP activity were determined at 610 nm with ε_610 nm_ = 2.2 × 10^3^ M^−1^·cm^−1^.

Overall, the enzymatic results were submitted to analysis of variance and means compared by Tukey test at 5% probability in the ASSISTAT software [28].

### 2.6. Phenotype Data Analysis

A cluster analysis was applied and a dendrogram of phenotype relatedness was constructed. The analysis was performed using the phenotype traits that showed at least high association with each *Trichoderma* species, such as: conidia shape, ornamentation, colour, length and width, and phialide length and base width, and presence of sterile hyphae and chlamydospore, and colony diameter on PDA at 30, 35 and 40 °C and SNA at 35 °C. The analysis was made using the hierarchical clustering with the complete linkage method and was performed with the statistical package jmp 8.0.2 for Macintosh (SAS Institute Inc., Cary, NC, USA).

## 3. Results and Discussion

### 3.1. Morphological and Molecular Identification

**Figure 1:**
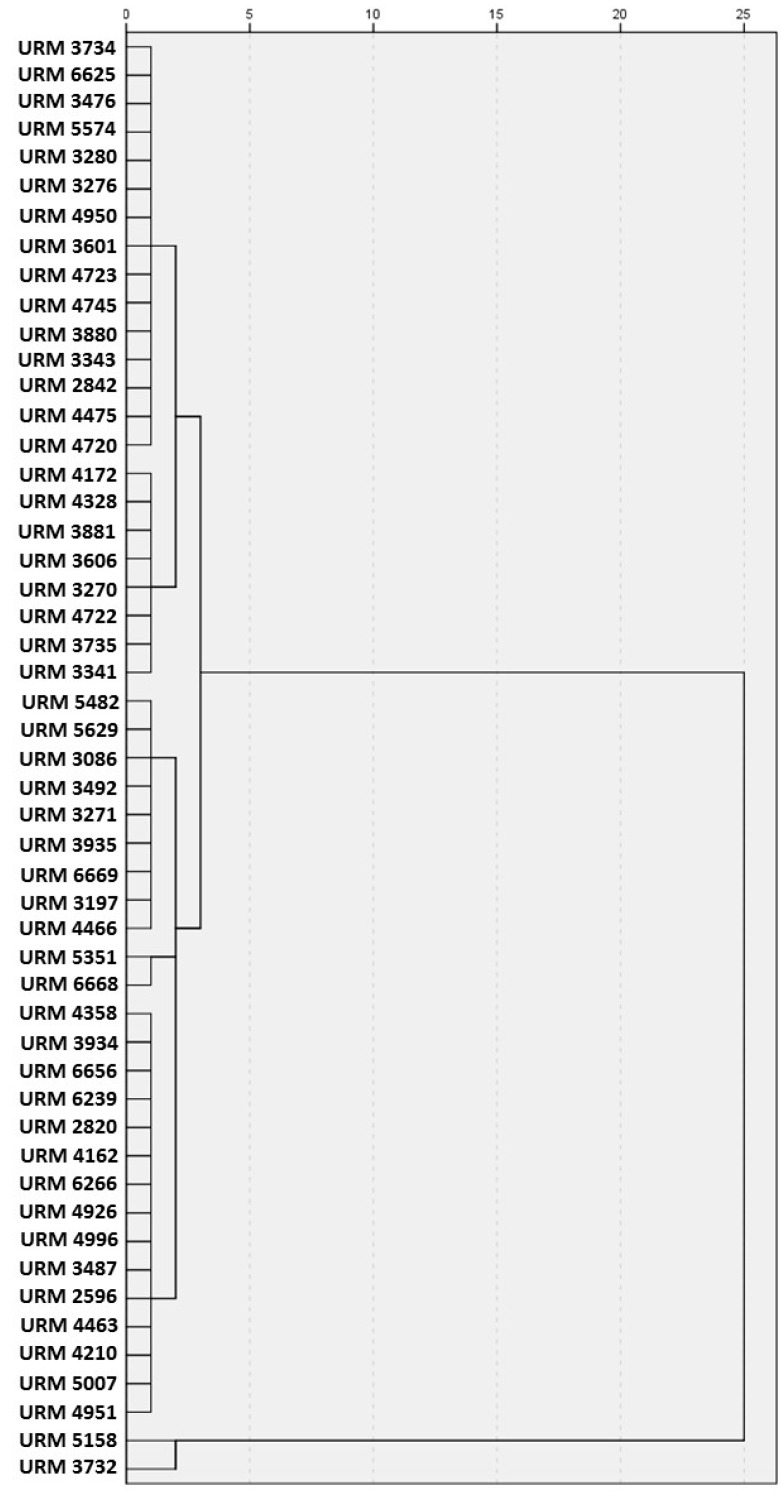
Dendrogram using the hierarchical cluster analysis (average linkage between groups) of relatedness among the strains of *Trichoderma* based on the morphological analysis data in Table 1.

Fifty-one fungal strains obtained from the Micoteca URM culture collection and belonging to the genus *Trichoderma* were evaluated. Data obtained from the morphological analysis (Table 1 and Figure 1) show a clear difficulty to cluster the strains by morphological traits when a cluster analysis was used. It is well known that, due to homoplasy (i.e., convergent evolution that creates analogous traits with similar form but were not present in the last common ancestor) of morphological characters, it is often impossible to discriminate species of *Trichoderma*. Notwithstanding this, for other fungal taxonomic groups, the statistical grouping of phenotype traits such as conidia shape, ornamentation and dimensions (length and wide) are normally very good data for species identification when combined with other traits [31,32].

It is well known that the overall range of variation in conidial dimensions in *Trichoderma* is not great. However, related species can often be differentiated by slight but consistent differences in size. The conidial surface appears smooth in most species in light microscope observations, although many species with apparently smooth-appearing conidia are delicately ornamented when examined by SEM. In most species, terminal phialides tend to be more elongate and narrower, and frequently more or less subulate. Chlamydospores are common in many species, although they tend to be uniformly globose or ellipsoidal, terminal and intercalary, smooth-walled, colourless, yellowish or greenish, and 6–15 um diameter in most species. Vegetative hyphae show few characters useful for identification.

This means that the identification based on the classical morphology techniques can be used as presumptive approach. However, to obtain a sound and reliable identification, molecular biology must be performed [33]. Consequently, in the present study the taxonomic information was revised throughout molecular biology analysis based on the 500 and 600 base pairs fragments obtained from the ITS1-5.8S-ITS2 rDNA and *tef1* gene regions, respectively.

**Figure 2:**
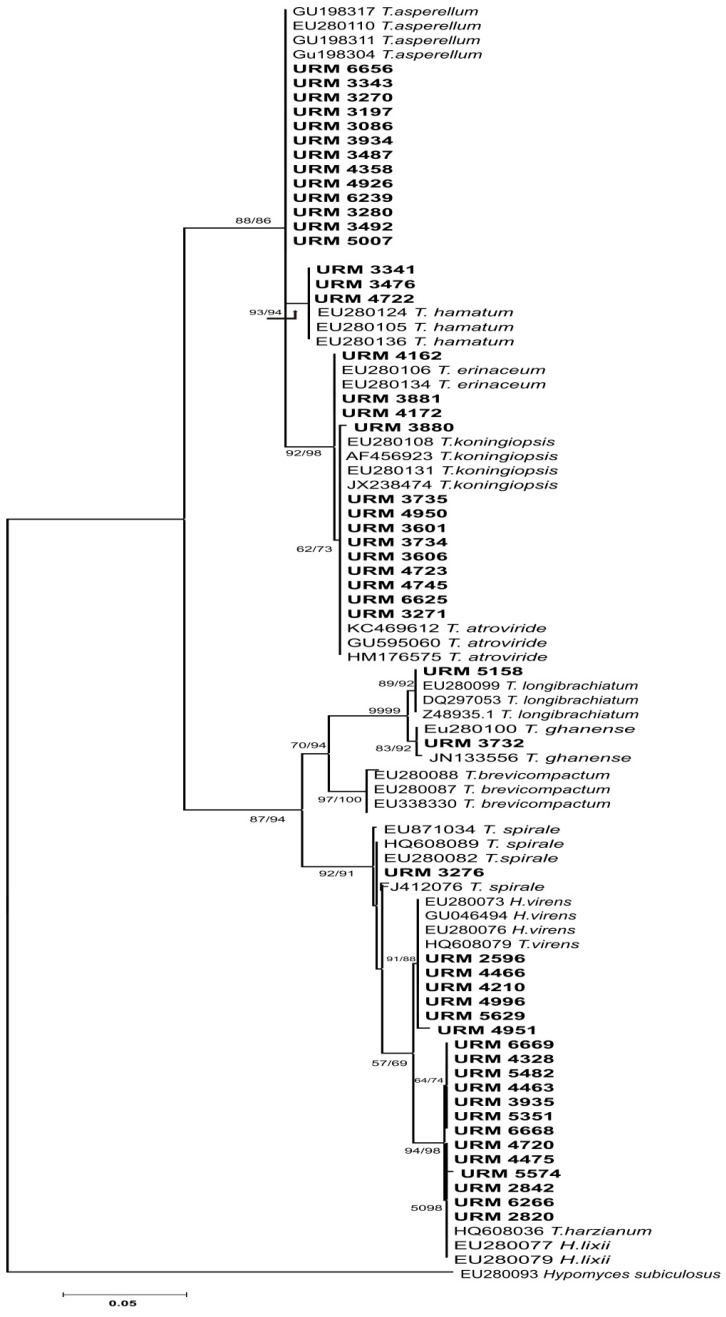
Molecular phylogenetic analysis of ITS1-5.8S-ITS2 for *Trichoderma* strains by maximum likelihood method. Indicated phylogenetic relationships were inferred with the maximum likelihood method based on the Kimura-2 parameter substitution model and 1000 bootstrap replicates conducted in MEGA5. Only those values with greater than 50% confidence are shown. Scale bar indicates nucleotide substitutions per site. *Hypomyces subiculosus* (EU280093 ITS sequence) was used as out group.

**Figure 3:**
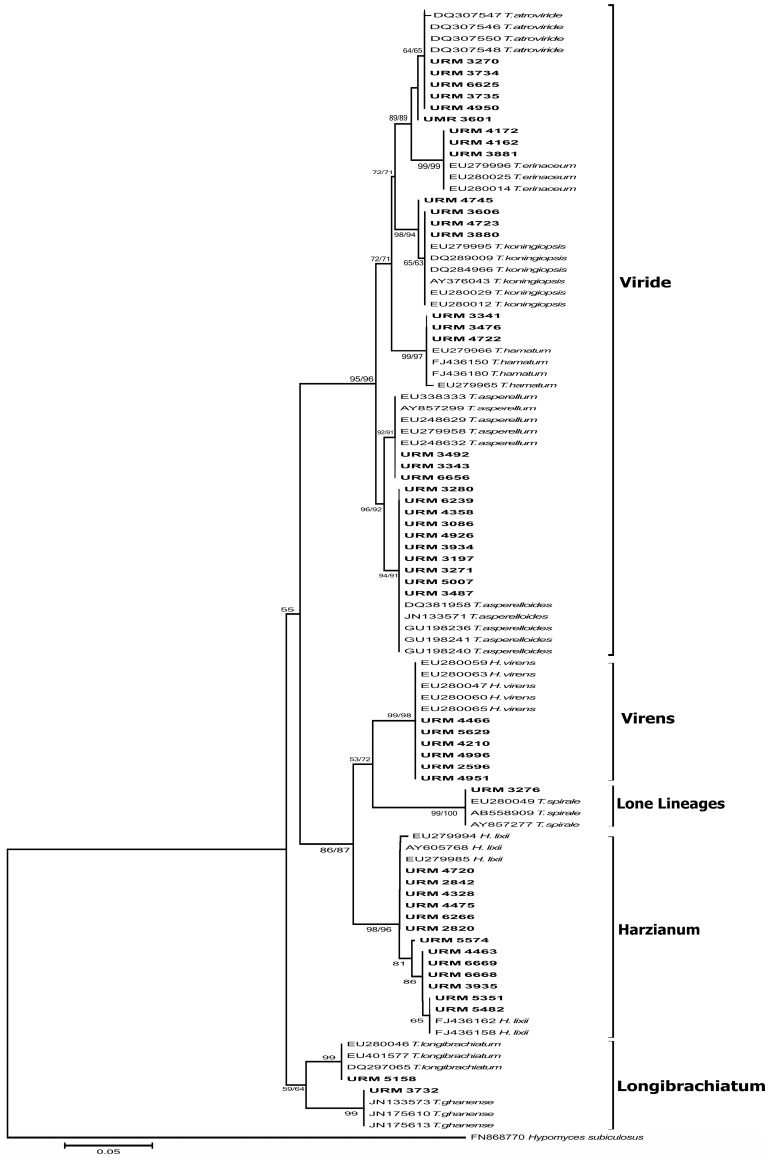
Molecular phylogenetic analysis of *tef1* for *Trichoderma* strains by maximum likelihood method. Indicated phylogenetic relationships were inferred with the maximum likelihood method based on the Kimura-2 parameter substitution model and 1000 bootstrap replicates conducted in MEGA5. Only those values with greater than 50% confidence are shown. Scale bar indicates nucleotide substitutions per site. *Hypomyces subiculosus* (FN868770 *tef1* sequence) was used as out group.

Through the phylogenetic analysis based on ITS region, inaccurate clades were observed and the groupings observed for some *Trichoderma* species were unclear (Figure 2). For instance, for cryptic species like *T. aspereloides* and *T. asperelum*, the ITS region was not discriminatory enough. In contrast, through the *tef1* gene analysis the species *T. aspereloides* and *T. asperelum* were well-delineated. In fact, in Figure 3, it is possible to observe six clades supported with bootstrap above 85%: Viride (*Trichoderma koningiopsis*, *T. erinaceum* and *T. atroviride*), Hamatum (*Trichoderma asperellum*, *T. asperelloides* and *T. hamatum*); Longibrachiatum (*Trichoderma longibrachiatum* and *T. ghanense*); Lone lineages (*Trichoderma spirale*), Virens (*Hypocrea virens*/*Trichoderma virens*), and Harzianum (*Hypocrea lixii*/*Trichoderma harzianum*).

Based on the former identification available in the records of the Micoteca URM culture collection, the 51 fungal isolates were distributed into six *Trichoderma* species, as shown in the Table 2. Based on the molecular biology analysis presented herein, it was possible to separate them into 11 different species: *Trichoderma asperelloides* (8 strains), *T. asperellum* (4), *T. atroviride* (6), *T. erinaceum* (3), *T. ghanense* (1), *T. hamatum* (4), *T. harzianum* (13), *T. koningiopsis* (4), *T. longibrachiatum* (1), *T. spirale* (1) and *T. virens* (6) (Table 2). Overall, only 10 out of 51 (ca. 20%) isolates retained their former identification, namely, *T. hamatum* (URM 3492), *T. harzianum* (URM 4475, 5482, 4720, 6266 and 4328) and *T. virens* (URM 4358, 3934, 3343 and 6656).

### 3.2. Screening of Indigo Carmine Decolourisation in Malt Broth

Among the 51 *Trichoderma* strains assessed for decolourisation of Indigo Carmine in MB, 18 of them presented over 70% decolourisation, which corresponds to 0.57 × 10^−2^ mM or less residual dye (Table 3). From these fungal strains, three presented values greater than 90% decolourisation: *T. atroviride* URM 3270, URM 3735 and URM 6625, which presented dye decolourisation of 96.86%, 94.61% and 93.57%, respectively. These results corroborate those previous obtained by Adnan et al. [14] for a strain of *T. atroviride* isolated from tree bark in a Malaysian forest. In that case, authors obtained similar results with 91.1% decolourisation for the dye Reactive Black 5.

In contrast, according to the data obtained in the present study, the strains *T. asperelloides* URM 3086, URM 3934 and URM 5007; *T. asperellum* URM 6656; *T. hamatum* URM 4722; *T. koningiopsis* URM 3606; and *T. virens* URM 4466 were not efficient in decolourising Indigo Carmine. These results were due to the fact strains belonging to these species require more time to be adapted on the polluted environment to become able to decolourise the effluent. Gajera et al. [34] obtained similar results when assessed the dye decolourisation capability of *T. viride* and *T. harzanium* strains isolated from contaminated rhizosphere with an industrial textile effluent in India. In this case, strains belonging to both species required a period longer than 12 days to decolourise completely reactive azo dyes, such as Red HE7B, Violet Reactive 5, Black Red-B, Dark Blue and Light Blue H2GP HEG.

### 3.3. Activity of Oxidase Enzymes

Fungal strains that presented decolourisation percentages of textile dye Indigo Carmine above 70% were selected for assessment of Lac, LiP and MnP activities (18 strains, Table 3). Based on the data obtained for Lac, LiP and MnP activities and after statistical analysis of averages, a significant difference between the means of LiP and MnP activities was observed (Table 3). In addition, the interaction among the averages of Lac, LiP and MnP indicated that all of the assessed fungal strains were better producers of LiP than the other two enzymes (Lac and MnP) (Table 3).

In a previous study performed by Adnan et al. [14], *T. atroviride* showed no activity for LiP and MnP. In contrast, in the present study, it was observed that the best LiP activity was obtained for the assays performed with *T. atroviride* URM 3735, which presented a production of 1307.0 U·L^−1^, followed by *T. harzianum* URM 2842 with an activity of 924.0 U·L^−1^ and *T. virens* URM 4996 with an activity of 844.0 U·L^−1^. In addition, herein, it was observed that for the MnP, the highest activity was obtained for the strain *T. erinaceum* URM 3881 (875.3 U·L^−1^), followed by *T. atroviride* URM 4950 and URM 3735, 680.0 U·L^−1^ and 617.3 U·L^−1^, respectively.

No significant difference between the averages of Lac activity was observed. However, the strain *T. virens* URM 4996 presented a Lac activity of 15.7 U·L^−1^ which is numerically greater than those observed for the other species assessed (Table 3). In a previous study, Adnan et al. [14] observed a Lac activity of 5.8 U·mL^−1^ after four days of experiment for a *T. atroviride* strain. Authors attested that Lac production was directly related to the degradation of textile dye Reactive Black 5. In the present work, the Lac production by the four fungal strains belonging to the species *T. atroviride* (URM 3270, 3735, 4950 and 3881) was assessed. After eight days of the experiment, Lac activities assessed were 1.3, 2.0, 8.3 and 2.3 U·L^−1^, respectively (Table 3).

Overall, based on the results obtained herein for activities of Lac, LiP and MnP, the following six strains were selected as the best producer of these oxidases and then used in the screening assays of effluent decolourisation: *Trichoderma asperellum* URM 4926, *T. atroviride* URM 3735, URM 4950, *T. erinaceum* URM 3881, *T. harzianum* URM 2842 and *T. virens* 4996 (Table 3).

### 3.4. Treatment of Real Textile Effluent

The six above mentioned *Trichoderma* strains, which were the best enzymatic producers were then selected to decolourise real textile effluent which is characterised by high organic load since the COD and BOD parameters are higher than the normal accepted for inland surface water disposal (COD < 250 mg·L^−1^ and BOD < 30 mg·L^−1^) (Table 4).

**Figure 4:**
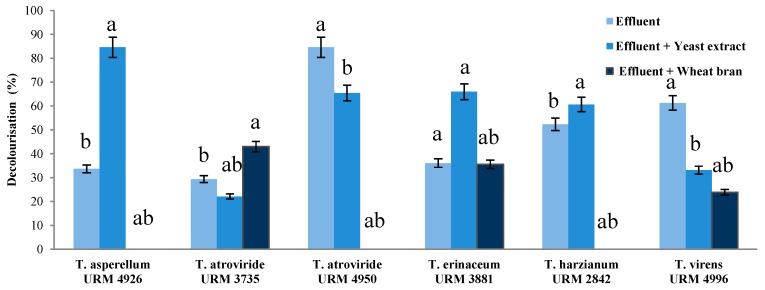
Decolourisation percentage of real textile effluent by the six *Trichoderma* strains best oxidases producers. Averages followed by the same letters do not differ by Friedman test at 5% probability.

Based on the data obtained for: (1) textile effluent; (2) textile effluent supplemented with 0.5% (*w*/*v*) wheat bran; and (3) textile effluent supplemented with 0.05% (*w*/*v*) extract yeast, for each strain, and after statistical analysis of averages, a significant difference between the means was observed (Figure 4). According to the results obtained, *T. atroviride* URM 4950, *T. erinaceum* URM 3881 and *T. virens* URM 4996 showed the best performance in decolourising of textile effluent (84.6%, 36.1% and 61.3%, respectively). In addition, *T. asperellum* URM 4926 (84.6%), *T. erinaceum* URM 3881(66%) and *T. harzianum* URM 2842 (60.6%) presented a good performance of decolourisation when textile effluent supplemented with extract yeast was used.

When the effluent was supplemented with wheat bran, the percentage of effluent decolourisation was higher for the strains *T. atroviride* URM 3735 (43.0%), *T. erinaceum* URM 3881 (35.6%) and *T. virens* URM 4996 (23.8%) (Figure 4).

According to Saravanakumar and Kathiresan [17], a large decolourisation rate (89%) of Malachite Green dye after 10 days of incubation of *T. harzianum* TSK8 (JQ809340) was observed when this strain was grown in supplemented medium with 5.8 mg·L^−1^ yeast extract. In addition, Adnan et al. [14] evaluated the effect of different sources of carbon and nitrogen in the decolourisation of textile dye Reactive Black 5 by *T. atroviride*. Authors observed that yeast extract was a good source of nitrogen for growth *T. atroviride* and to stimulate it to decolourise Reactive Black 5 up to 91.1%. Furthermore, authors evaluated dye decolourisation by using ammonium nitrate and ammonium chloride and, in contrast, observed only a decolourisation rate of 29.1% and 71.7%, respectively.

## 4. Conclusions

The Brazilian *Trichoderma* asset preserved at the Micoteca URM culture collection was re-identified and made available in the culture collection e-catalogue to be further explored from the biotechnologically point of view. In the specific case of dye decolourisation screening using real textile effluent, the strain *T. atroviride* URM 4950 gave the best results and can be considered in a scale-up process at industrial level. This strain is herein indicated to be used as a major target for future studies related with the treatment of industrial textile effluent and bioremediation of environments contaminated with dyes. Overall, the data obtained suggest that the dye decolourisation in real textile effluent is not a one-step process. More than one oxidative enzyme can be involved in this complex process. The contribution of these enzymes for decolourisation of textile effluent differs from fungus to fungus. The potential of *Trichoderma* strains in decolourising real textile dye present in textile effluent and the production of the oxidative enzymes Lac, LiP and MnP were successfully demonstrated. Finally, in this study, the strains available through Micoteca URM culture collection are identified according the current state-of-the-art and better characterised to underpinning life sciences and biotechnology.

## Figures and Tables

**Table 1:** Morphological traits of 51 *Trichoderma* strains from Micoteca URM culture collection.

URM	Conidia					Phialide		Sterile Hyphae	Chlamyd.	PDA (mm)			SNA (mm)
	Shape ^1^	Ornament ^2^	Colour ^3^	Length (μm)	Width (μm)	Length (μm)	Base Width (μm)			30 °C	35 °C	40 °C	35 °C
3280	GL/SGL	SW	G	2.8–3.4	2.2–2.7	10.2–11.9	1.8–2.0	−	+	67.0	9.0	Ø	7.0
6239	GL/SGL	SW	G	2.8–3.4	3.2–3.6	8.5–10.2	1.8–2.0	−	−	77.0	24.0	Ø	16.0
3086	GL/SGL	SW	G	2.8–3.4	2.2–2.7	10.2–11.9	1.8–2.0	−	+	51.0	31.0	Ø	23.0
3487	GL/SGL	SW	G	2.8–3.4	2.2–2.7	10.2–11.9	1.8–2.0	−	+	74.0	28.0	Ø	24.0
3271	GL/SGL	SW	G	2.8–3.4	3.2–3.6	8.5–10.2	1.8–2.0	−	−	65.0	26.0	Ø	15.0
5007	SGL/O	SW	G	2.8–3.4	2.7–3.2	8.5–10.2	2.0–2.6	−	−	78.0	44.0	Ø	23.0
4358	GL/SGL	SW	G	3.5–4.1	2.2–2.7	10.2–11.9	1.8–2.0	−	+	76.0	25.0	Ø	20.0
3934	GL/SGL	SW	G	3.5–4.1	2.2–2.7	10.2–11.9	1.8–2.0	−	+	75.0	23.0	Ø	21.0
3343	GL/SGL	SW	G	2.8–3.4	2.2–2.7	8.5–10.2	2.0–2.6	−	−	73.0	14.0	Ø	6.0
6656	SGL/O	SW	G	2.8–3.4	2.2–2.7	8.5–10.2	2.0–2.6	−	+	77.0	22.0	Ø	18.0
4926	GL/SGL	SW	G	2.8–3.4	3.2–3.6	8.5–10.2	1.8–2.0	+	+	71.0	32.0	Ø	27.0
3197	SGL/O	SW	G	2.8–3.4	2.2–2.7	10.2–11.9	2.6–3.1	−	+	52.0	24.0	Ø	18.0
3735	GL/SGL	S	G	3.5–4.1	2.7–3.2	8.5–10.2	2.6–3.1	−	−	44.0	Ø	Ø	Ø
3734	GL/SGL	S	G	3.5–4.1	2.7–3.2	8.5–10.2	2.6–3.1	−	−	71.0	Ø	Ø	Ø
6625	GL/SGL	S	G	2.8–3.4	2.7–3.2	8.5–10.2	1.8–2.0	−	−	69.0	Ø	Ø	Ø
4950	GL/SGL	S	G	3.5–4.1	2.7–3.2	8.5–10.2	2.6–3.1	−	−	77.0	Ø	Ø	Ø
3601	GL/SGL	S	G	3.5–4.1	2.7–3.2	8.5–10.2	2.6–3.1	−	−	80.0	Ø	Ø	Ø
3270	GL/SGL	S	G	3.5–4.1	2.7–3.2	8.5–10.2	2.6–3.1	−	−	40.0	Ø	Ø	Ø
4162	E	S	G	4.2–4.8	2.7–3.2	6.8–8.5	1.8–2.0	−	+	73.0	20.0	Ø	17.0
4172	E	S	G	4.2–4.8	3.2–3.6	6.8–8.5	1.8–2.0	−	+	46.0	9.0	Ø	8.0
3881	E	S	G	4.2–4.8	3.2–3.6	6.8–8.5	1.8–2.0	−	+	50.0	14.0	Ø	11.0
3732	E	S	G	4.9–5.5	2.7–3.2	5.1–6.8	2.0–2.6	−	−	75.0	75.0	56.0	77.0
3341	SGL/O	S	G	3.5–4.1	2.7–3.2	10.2–11.9	2.0–2.6	+	+	54.0	Ø	Ø	Ø
3476	SGL/O	S	G	3.5–4.1	2.7–3.2	10.2–11.9	2.0–2.6	+	+	63.0	Ø	Ø	Ø
3492	SGL/O	S	G	2.8–3.4	2.7–3.2	10.2–11.9	1.8–2.0	+	+	64.0	25.0	Ø	9.0
4722	SGL/O	S	G	3.5–4.1	2.7–3.2	10.2–11.9	2.0–2.6	+	+	42.0	Ø	Ø	Ø
5351	SGL/O	S	G	2.4–2.7	1.8–2.2	8.5–10.2	1.8–2.0	+	+	75.0	35.0	Ø	36.0
6668	SGL/O	S	G	2.8–3.4	2.2–2.7	6.8–8.5	1.8–2.0	−	+	79.0	44.0	Ø	38.0
3935	SGL/O	S	G	2.4–2.7	1.8–2.2	10.2–11.9	2.0–2.6	−	−	58.0	20.0	Ø	14.0
5574	SGL/O	S	G	2.8–3.4	1.8–2.2	6.8–8.5	1.8–2.0	+	−	65.0	7.0	Ø	6.0
4463	SGL/O	S	G	2.8–3.4	1.8–2.2	8.5–10.2	1.8–2.0	−	−	78.0	29.0	Ø	29.0
2842	SGL/O	S	G	2.8–3.4	2.2–2.7	10.2–11.9	2.0–2.6	−	−	71.0	13.0	Ø	10.0
6669	SGL/O	S	G	2.4–2.7	1.8–2.2	10.2–11.9	2.0–2.6	−	−	58.0	17.0	Ø	12.0
4475	SGL/O	S	G	2.4–2.7	1.8–2.2	10.2–11.9	2.0–2.6	−	−	68.0	17.0	Ø	13.0
5482	SGL/O	S	G	2.4–2.7	1.8–2.2	10.2–11.9	2.0–2.6	−	−	57.0	31.0	Ø	28.0
4720	GL/SGL	S	G	2.4–2.7	2.2–2.7	8.5–10.2	2.0–2.6	−	−	64.0	15.0	Ø	11.0
6266	SGL/O	S	G	2.4–2.7	1.8–2.2	5.1–6.8	1.8–2.0	−	+	79.0	28.0	Ø	11.0
4328	SGL/O	S	G	2.4–2.7	1.8–2.2	10.2–11.9	2.0–2.6	−	−	50.0	8.0	Ø	8.0
2820	SGL/O	S	G	2.8–3.4	1.8–2.2	6.8–8.5	2.0–2.6	−	−	74.0	23.0	Ø	19.0
3606	GL/SGL	S	G	3.5–4.1	2.7–3.2	13.6–15.2	2.0–2.6	−	−	54.0	12.0	Ø	3.0
4723	E	S	G	3.5–4.1	1.8–2.2	6.8–8.5	1.8–2.0	−	−	76.0	8.0	Ø	15.0
4745	E	S	G	3.5–4.1	1.8–2.2	6.8–8.5	1.8–2.0	−	−	74.0	10.0	Ø	17.0
3880	E	S	G	3.5–4.1	1.8–2.2	6.8–8.5	1.8–2.0	−	−	72.0	11.0	Ø	13.0
5158	E	S	G	3.5–4.1	2.2–2.7	10.2–11.9	2.6–3.1	−	+	83.0	83.0	31.0	80.0
3276	OB	S	G	3.5–4.1	2.7–3.2	5.1–6.8	2.6–3.1	+	+	67.0	9.0	Ø	Ø
5629	SGL/O	S	G	3.5–4.1	2.2–2.7	13.6–15.2	1.8–2.0	−	+	56.0	28.0	Ø	27.0
4210	SGL/O	S	G	3.5–4.1	2.7–3.2	13.6–15.2	2.0–2.6	−	−	82.0	27.0	Ø	24.0
4466	GL/SGL	S	G	3.5–4.1	2.7–3.2	10.2–11.9	2.0–2.6	−	+	53.0	21.0	Ø	20.0
4951	GL/SGL	S	G	3.5–4.1	2.7–3.2	10.2–11.9	2.0–2.6	+	+	66.0	43.0	Ø	16.0
4996	GL/SGL	S	G	4.2–4.8	3.2–3.6	8.5–10.2	2.6–3.1	−	+	69.0	35.0	Ø	25.0
2596	SGL/O	S	W/Y	3.5–4.1	2.7–3.2	13.6–15.2	2.6–3.1	−	+	73.0	28.0	Ø	28.0

^1^ E, Ellipsoidal; GL, Globose; SGL, Subglobose; O, Ovoid; OB, Oblong. ^2^ S, Smooth; SW, Slightly Warty. ^3^ G, Green; W/Y, White to Yellow. Symbols: −, Absent; +, Present; Ø, Without growth.

**Table 2:** Re-identification of bio-resources from Micoteca URM culture collections after morphological and molecular characterisation.

URM	Revised Identification	Original Identification	Deposit Year	Substrate	Geographical Origin (Brazil)
3280	*T. asperelloides*	*T. harzianum*	1992	Soil	Paraná
6239	*T. asperelloides*	*T. harzianum*	2010	Soil	Pernambuco
3086	*T. asperelloides*	*T. harzianum*	1989	Leaf of *R. gardenioides*	São Paulo
3487	*T. asperelloides*	*T. koningii*	1994	Birds faeces	Pernambuco
3271	*T. asperelloides*	*T. koningii*	1992	Unknown	São Paulo
5007	*T. asperelloides*	*T. virens*	2005	Soil	Pernambuco
4358	*T. asperelloides*	*T. virens*	2001	Soil	Pernambuco
3934	*T. asperelloides*	*T. virens*	1997	Water	Pernambuco
3343	*T. asperellum*	*T. aureoviride*	1993	Unknown	Pernambuco
6656	*T. asperellum*	*T. hamatum*	2012	Soil with textile effluent	Pernambuco
4926	*T. asperellum*	*T. harzianum*	2005	Clay soil	Pernambuco
3197	*T. asperellum*	*T. harzianum*	1990	Amazonian nuts	Pará
3735	*T. atroviride*	*T. aureoviride*	1997	Lake water	Pernambuco
3734	*T. atroviride*	*T. aureoviride*	1997	Lake water	Pernambuco
6625	*T. atroviride*	*T. aureoviride*	2012	Soil of agroforestry	Pernambuco
4950	*T. atroviride*	*T. harzianum*	2005	Soil	Pernambuco
3601	*T. atroviride*	*T. harzianum*	1995	Unknown	Paraná
3270	*T. atroviride*	*T. harzianum*	1992	Unknown	São Paulo
4162	*T. erinaceum*	*T. aureoviride*	1999	Rhizosphere of sunflower	Pernambuco
4172	*T. erinaceum*	*T. harzianum*	1999	Rhizosphere of sunflower	Pernambuco
3881	*T. erinaceum*	*T. koningii*	1997	Soil of *P. edulis* culture	Pernambuco
3732	*T. ghanense*	*T. koningii*	1997	Lake water	Pernambuco
3341	*T. hamatum*	*T. aureoviride*	1992	Rhizosphere of *V. herbacea*	São Paulo
3476	*T. hamatum*	*T. aureoviride*	1993	Rhizosphere of *V. herbacea*	São Paulo
3492	*T. hamatum*	*T. hamatum*	1994	Birds faeces	Pernambuco
4722	*T. hamatum*	*T. harzianum*	2003	Garden of *A. cephalotes*	Alagoas
5351	*T. harzianum*	*T. aureoviride*	2006	Sugarcane	Unknown
6668	*T. harzianum*	*T. aureoviride*	2012	Soil with textile effluent	Pernambuco
3935	*T. harzianum*	*T. aureoviride*	1997	River water	Pernambuco
5574	*T. harzianum*	*T. aureoviride*	2007	Mangrove sediment	Pernambuco
4463	*T. harzianum*	*T. aureoviride*	2002	Sea water	Pernambuco
2842	*T. harzianum*	*T. aureoviride*	1986	Unknown	Pernambuco
6669	*T. harzianum*	*T. aureoviride*	2012	Soil with textile effluent	Pernambuco
4475	*T. harzianum*	*T. harzianum*	2002	Beach sand	Pernambuco
5482	*T. harzianum*	*T. harzianum*	2007	Unknown	São Paulo
4720	*T. harzianum*	*T. harzianum*	2003	Garden of *A. cephalotes*	Alagoas
6266	*T. harzianum*	*T. harzianum*	2010	Soil of agroforestry	Pernambuco
4328	*T. harzianum*	*T. harzianum*	2001	Soil of mining	Bahia
2820	*T. harzianum*	*T. viride*	1985	Sugarcane bagasse	Alagoas
3606	*T. koningiopsis*	*T. hamatum*	1995	Unknown	Paraná
4723	*T. koningiopsis*	*T. koningii*	2003	Garden of *A. cephalotes*	Alagoas
4745	*T. koningiopsis*	*T. koningii*	2003	Garden of *A. cephalotes*	Alagoas
3880	*T. koningiopsis*	*T. koningii*	1997	Soil of *P. edulis* culture	Pernambuco
5158	*T. longibrachiatum*	*T. aureoviride*	2005	Cement	Pernambuco
3276	*T. spirale*	*T. hamatum*	1991	sorghum	Pernambuco
5629	*T. virens*	*T. hamatum*	----	Unknown	Paraná
4210	*T. virens*	*T. virens*	1999	Rhizosphere of sunflower	Pernambuco
4466	*T. virens*	*T. virens*	2002	Sea water	Pernambuco
4951	*T. virens*	*T. virens*	2005	Soil	Pernambuco
4996	*T. virens*	*T. virens*	2005	Soil	Pernambuco
2596	*T. virens*	*T. viride*	1980	Unknown	Pernambuco

**Table 3:** The 18 *Trichoderma* URM strains that presented decolourisation percentages of Indigo Carmine above 70% and related oxidase enzyme activities (U·L^−1^) after eight days of incubation.

Species	URM	Lac	LiP	MnP
*T. asperelloides*	3280	1.7 ^aC^	760.0 ^cdA^	379.3 ^defB^
*T. asperellum*	4926	8.3 ^aC^	517.3 ^fghA^	366.0 ^defgB^
*T. atroviride*	3270	2.0 ^aC^	758.0 ^cdA^	394.0 ^defB^
*T. atroviride*	3735	8.3 ^aC^	1307.0 ^aA^	617.3 ^bcB^
*T. atroviride*	4950	2.3 ^aB^	750.0 ^cdA^	680.0 ^bA^
*T. atroviride*	6625	1.3 ^aB^	692.0 ^deA^	90.7 ^jlB^
*T. erinaceum*	3881	10.0 ^aC^	378.7 ^iB^	875.3 ^aA^
*T. ghanense*	3732	1.7 ^aC^	766.3 ^cdA^	298.0 ^fghB^
*T. hamatum*	3476	1.0 ^aC^	593.3 ^efgA^	92.7 ^jlB^
*T. harzianum*	2842	2.3 ^aC^	924.0 ^bA^	176.0 ^hijB^
*T. harzianum*	3935	1.3 ^aC^	480.0 ^ghiA^	143.3 ^ijB^
*T. harzianum*	4328	2.7 ^aB^	378.0 ^iA^	442.7 ^deA^
*T. harzianum*	4463	1.7 ^aC^	758.7 ^cdA^	232.0 ^ghiB^
*T. harzianum*	5482	2.7 ^aC^	631.3 ^defA^	369.3 ^defB^
*T. harzianum*	6668	3.0 ^aB^	440.0 ^hiA^	0.0 ^lB^
*T. koningiopsis*	3880	7.7 ^aC^	645.3 ^defA^	146.0 ^ijB^
*T. virens*	4996	15.7 ^aC^	844.0 ^bcA^	329.3 ^efgB^
*T. virens*	5629	1.7 ^aC^	612.7 ^efgA^	500.0 ^cdB^

Averages values followed by the same superscript letters, lower case (within column) and capital (within row), do not differ by Tukey test at 5% probability.

**Table 4:** Physical-chemical analyses of the effluent obtained from the textile manufacture located at the municipality of Toritama, Pernambuco, Brazil.

COD (mg O_2_·L^−1^)	BOD (mg O_2_·L^−1^)	Colour (HAZEN)	Turbidity (NTU)	pH	SD (mL·L^−1^)
3192.5	54.1	448.0	0.19	5.11	7.0
